# What to Expect When Switching to a Biosimilar: A US Healthcare Professional’s Perspective

**DOI:** 10.1093/crocol/otae063

**Published:** 2024-11-25

**Authors:** Juanita Belton, Nicholas D’Apice

**Affiliations:** Gastroenterology Department, Boston Medical Center, Boston, MA, USA; Ambulatory Care Clinical Pharmacy, Boston Medical Center, Boston, MA, USA

**Keywords:** biologic, biosimilar, drug switching

## Abstract

As biosimilars become more available, many patients with inflammatory bowel disease may experience having their treatment switched from a reference product to a biosimilar. In this communication, a physician assistant and a pharmacist discuss the patient experience when switching to a biosimilar.

## Graphical Abstract

**Graphical Abstract:**
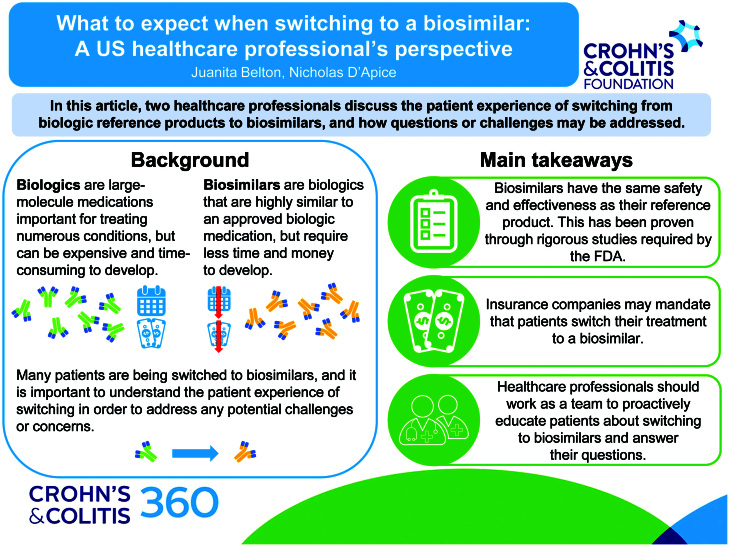

## Introduction

Biologic medications, also known as biologics, are large-molecule medications made in living cells or tissue.^1,2^ They have become increasingly important for treating a number of conditions, including cancer and autoimmune conditions (eg, rheumatoid arthritis, psoriasis, and inflammatory bowel disease).^3,4^ However, biologics can be very expensive to make and use, often placing a major financial burden on the healthcare system.^5^ Biosimilars are biologics designed to have identical properties to an existing biologic, known as the reference product. While biosimilars may have some small differences in their composition compared with their reference products, they are proven to have the same safety and efficacy as the reference product.^1,2^ The intention of biosimilars is to reduce medication costs and ensure that no single manufacturer of biologic drugs dominates the healthcare market.^5,6^ To expedite their approval, the US Food and Drug Administration (FDA) introduced a specialized approval process for biosimilars in 2010 with the passage of the Biologics Price Competition and Innovation Act. Since then, 53 different biosimilar drugs have been approved to treat patients in the United States as of April 2024 (Table 1).^7^ Many patients have already experienced being switched from a reference product to a biosimilar as they become increasingly available to reduce costs.^8^ As switching to biosimilars becomes more common, it is important to address any concerns or questions patients may have about the switch. In this article, two healthcare professionals discuss the switching process, what questions or challenges commonly arise, and how they can be addressed.

**Table 1.** Select biosimilars approved by the FDA.^7^

Reference product	Biosimilar name	Approval date
Humira (adalimumab)	Simlandi (adalimumab-ryvk)	February 2024
Humira (adalimumab)	Yuflyma (adalimumab-aaty)	May 2023
Humira (adalimumab)	Idacio (adalimumab-aacf)	December 2022
Humira (adalimumab)	Yusimry (adalimumab-aqvh)	December 2021
Humira (adalimumab)	Hulio (adalimumab-fkjp)	July 2020
Humira (adalimumab)	Abrilada (adalimumab-afzb)	November 2019
Humira (adalimumab)	Hadlima (adalimumab-bwwd)	July 2019
Humira (adalimumab)	Hyrimoz (adalimumab-adaz)	October 2018
Humira (adalimumab)	Cyltezo (adalimumab-adbm)	August 2017
Humira (adalimumab)	Amjevita (adalimumab-atto)	September 2016
Remicade (infliximab)	Avsola (infliximab-axxq)	December 2019
Remicade (infliximab)	Ixifi (infliximab-qbtx)	December 2017
Remicade (infliximab)	Renflexis (infliximab-abda)	May 2017
Remicade (infliximab)	Inflectra (infliximab-dyyb)	April 2016

## Discussion

### How Do You Describe Biosimilars to Patients?

**Figure 1.**
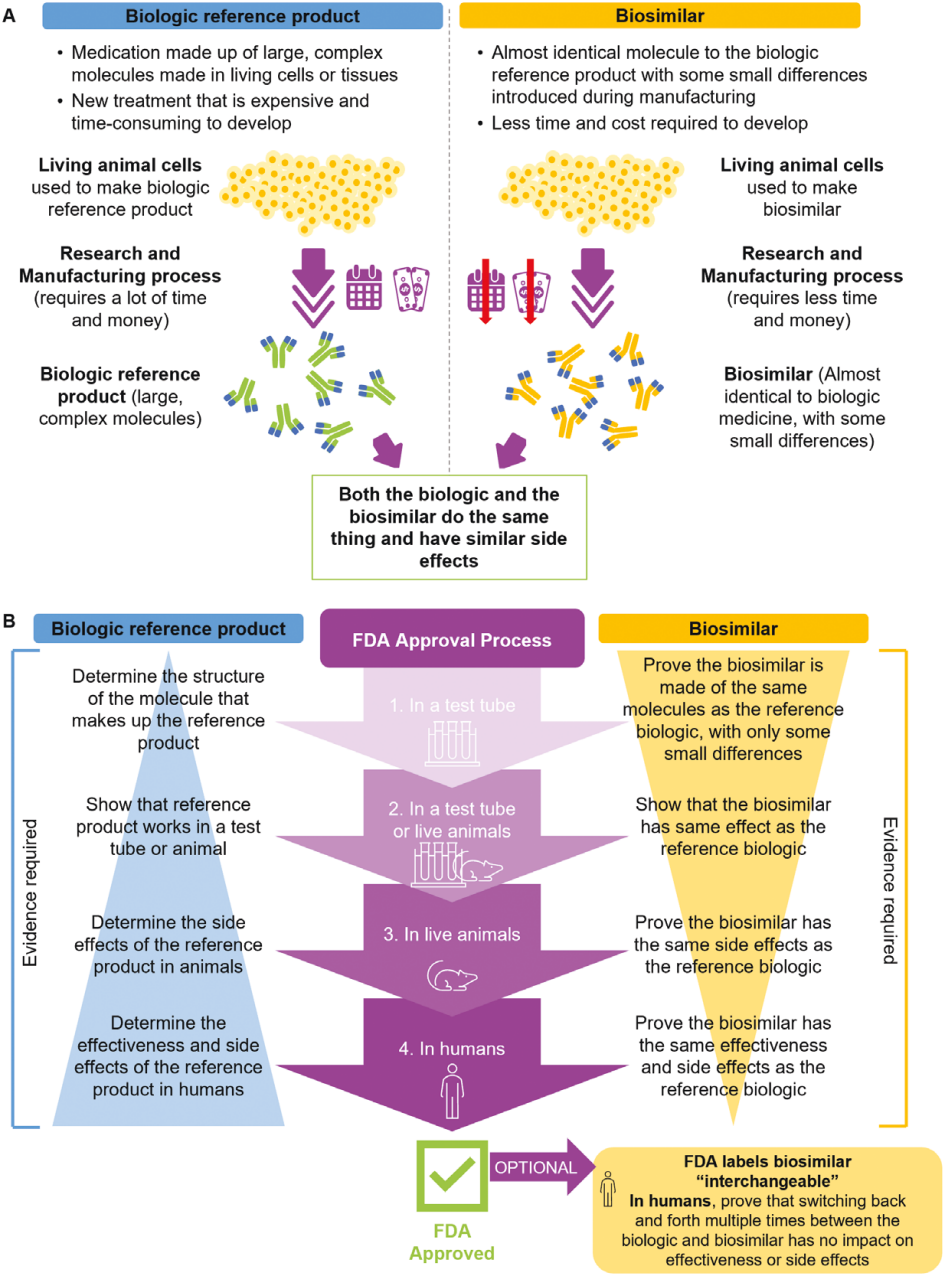
Plain language explanation of biosimilars. A, Biologic reference products vs. biosimilars. B, FDA approval process of biologic reference products and biosimilars.

When first describing biosimilars to patients, we explain that biologics are a type of medication made up of large, complex molecules that are manufactured in living cells or tissues (Figure 1A).^1,2^ Biosimilars are biologics that are highly similar to the original reference biologic, but because they are produced using living cells, it is impossible to create exact copies. Therefore, some small differences may be introduced during manufacturing.^2^ During initial discussions, some patients may ask if a biosimilar is the same as a generic product. When this comes up, we briefly explain that generic medicines are exact copies of small-molecule drugs that are manufactured chemically, while biosimilars may have small inherent changes given that they are manufactured from living cells. While a biosimilar cannot be considered exactly the same molecule as its reference product in the same way as a generic, the FDA requires a rigorous testing process to ensure that biosimilars produce the same therapeutic effects and have the same safety profile and side effects as the reference product (Figure 1B).^2^ It should be noted that in the USA, there is a further level of regulatory designation for biosimilars called interchangeable biosimilars. An interchangeable biosimilar undergoes an additional study to confirm that the effectiveness and safety profile are the same in patients who are switched multiple times between the biosimilar and the reference product as in patients who remain on the reference product. Where state laws allow, an interchangeable biosimilar can be substituted for its reference product by a pharmacist without the need for a new prescription from a provider.^9^

### What Happens When a Patient Switches to a Biosimilar?

**Figure 2.**
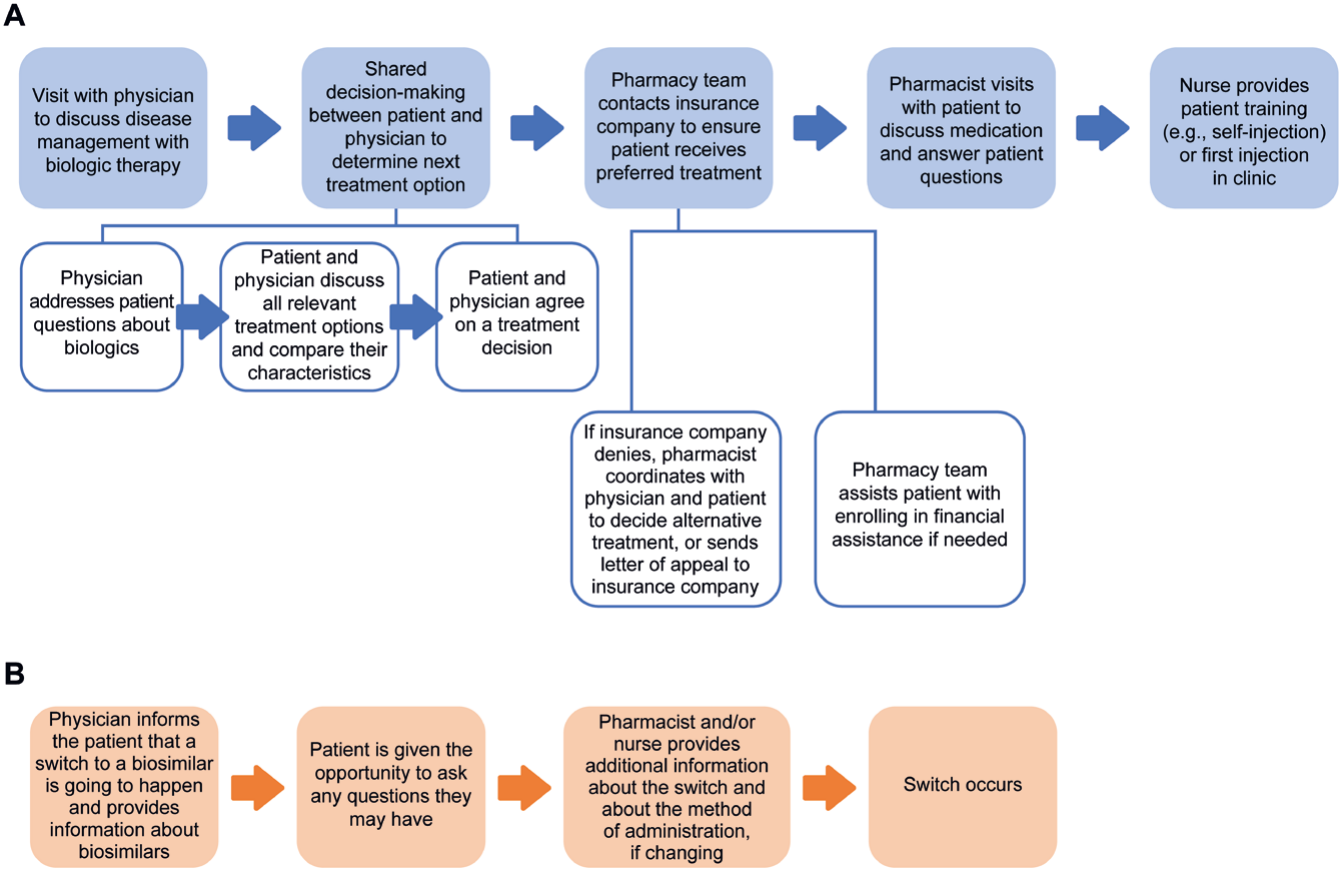
A, Patient journey initiating biologic treatment. B, Ideal patient journey switching to a biosimilar.

In our practice, when a patient is switched from a biologic to a biosimilar, our pharmacy team will inform the patient of the switch and answer any questions they have. Right now, there is a general lack of readily available information about biosimilars for both patients and healthcare professionals, and this needs to be addressed. Most patients have never heard of biosimilars, and they are therefore unlikely to proactively ask about biosimilars. It is important that healthcare professionals give patients the information they need upfront, to help them understand what biosimilars are and why they are recommended. It can be difficult to offer this information to patients when they are first started on a biologic, as there is already a lot of information the patient will need to take in at this time (Figure 2). This may mean there is not enough time to provide detailed information about biosimilars as well. However, it is crucial that a patient is educated about biosimilars and switching before the switch occurs.

From the start of the switch, most patients want to know why the change is occurring and what, if anything, will be different about the medication. This includes potential differences in effectiveness and side effects. We emphasize to patients that biosimilars have both the same efficacy and side effect profile as their reference product. We explain that the FDA requires rigorous studies for every biosimilar to prove this before it is approved (Figure 1B). Indeed, most patients are reassured when they find that they do not have any additional side effects when switching to a biosimilar, as new side effects are very uncommon. Another possibility is that the body’s immune system will react differently to the biosimilar, resulting in the development of antidrug antibodies.^10^ However, this has not typically been an issue or a concern for most patients.

Additional patient questions may be around the method of administration. When patients switch from a biologic to a biosimilar, they may have preferences for whether they receive the medication intravenously (IV) at the care facility or via subcutaneous (SC) self-injection at home and whether they use prefilled syringes or autoinjectors. Often, we find patients prefer to maintain the same method of administration as their original medication. It can help patients to accept the switch if both the reference product and the biosimilar are administered in the same manner. In the case that administration methods are switched from IV to SC, a nurse at our practice will provide information to patients on how to administer the SC medication on their own.

### When Are Patients Switched to a Biosimilar?

Patients are switched to a biosimilar when it is more cost-effective for the insurance company than the reference product. If patients are confused about why the switch is occurring, it is important to explain that there is a cost–benefit and that the biosimilar has the same efficacy and side effects as their previous medication. Therefore, we will tell patients that the medication is a biosimilar to the initial biologic and that there are no clinically meaningful differences.

While for most patients there would be no reason to avoid switching to a biosimilar, some providers may still be hesitant to make the switch, especially if it seems the patient was doing well on the reference product. In these instances, it may be beneficial for providers to have more education and awareness about biosimilars, in addition to the patients.

### What is the Patient Experience Like with Biologics/Biosimilars?

The typical patient journey with biologics begins when the patient presents with symptoms and gets a confirmed diagnosis from their physician. Following this, the patient will come back to the clinic for a follow-up meeting with their physician to receive more information about the disease, what the risks are, and how it can be managed (Figure 2A). Typically, we will discuss infusion, injectable, and oral medications. We also talk about side effects and expectations for when and how well the treatment will work.

When starting biologic treatment, most of the questions patients ask are about the side effects and effectiveness of the biologic, as these are typically their primary concerns. We try to reassure patients that serious side effects are rare and that biologics are generally safe and effective, and we discuss patient/peer support groups that are available to them for more information and support. If a patient does experience side effects that are similar to those experienced with the reference product, such as an allergic reaction, we discuss pretreatment with antihistamines or steroids or switching drug classes altogether. In rare cases where a new side effect occurs that cannot be prevented or mitigated, patients may have the option to switch back to the reference product. At this time, patients may also learn that they will be immunosuppressed when receiving certain biologics, and they often want to know how that will impact their lives. Will they be able to travel? Will they have to completely socially isolate themselves? In these cases, we educate patients about proper monitoring while on biologic therapy, including the need for hepatitis B and tuberculosis screenings. It is important to note that some patients may be the first member of their family to have the disease and are therefore likely to have a lot more questions relative to other patients.

At this stage in the patient’s journey, treatment options are a main point of discussion. This involves a conversation with the patient to determine what treatment plan will work best for them. We start by presenting all the treatment options to the patient. We then ask for patients’ preferences and inquire about their lifestyles to help determine the most feasible choice. For example, if they are older, they may prefer IV infusions because they do not want to self-administer the medication at home, though if they are younger, home injection may be preferable due to busy schedules. At this point, we will make a treatment recommendation, but inform the patient that their insurance company will likely have a preferred treatment. However, we assure the patients that we will do our best to advocate for them to receive the treatment of their choice. To obtain prior authorization for switching to a particular biosimilar, we have a dedicated pharmacy liaison technician who will reach out to the insurance company and let them know we want the patient to start this biosimilar. The insurance company will inform us of which biosimilar they prefer to cover. Advocating for patients to the insurance companies is the job of the pharmacy team and may consist of initiating appeals, writing letters of medical necessity, or having a peer-to-peer conversation with a provider at the insurance company.

The pharmacy staff also plays an important educational role when a patient is switched from a biologic to a biosimilar (Figure 2B). Pharmacists help to educate the patient about why they are switching; what the biosimilar is; how it will work; and what, if anything, will change (eg, administration method). The information provided by the pharmacist augments the information provided by the physician.

### What Are Some of the Challenges With Switching to Biosimilars?

So far, there have been very few challenges with switching to biosimilars. We are successfully able to reassure most patients that there will be no changes to efficacy and side effects, and most patients do very well on biosimilar treatment. Occasionally, a patient may switch to a biosimilar and report that they feel different than when they were receiving the reference product. These patients may report that they felt better when on the reference product, even though their lab results are not different, there is no active inflammation, and they have no new symptoms. This effect may be psychological and could be improved by providing patients with more information and education about biosimilars and how any side effects should be the same as those of the reference product.

If patients have not been made aware that a switch between treatment products is going to be made, they might get confused and think they are receiving the incorrect medication. In some cases, the formulation or the route of administration (IV vs. SC) of the biosimilar may be different from the reference product, and therefore, the brand or label reads differently. Understandably, patients are wary of being switched to a different medication without being informed it is happening. Thus, proactive outreach to patients to explain any switch before it happens is crucial.

### How Can Patients Best Advocate for Themselves and Their Treatment Plans?

Patients should always be encouraged to ask their physician or pharmacist any questions they have about their condition and treatment. Unfortunately, patients sometimes assume that doctors know everything and should not be questioned. However, if a patient has any discomfort or concerns with their treatment plan, they should be encouraged to speak up. Patients should feel free to ask any question. A healthcare professional’s goal is to work with the patient and figure out what treatment will be best for them. Therefore, the patient should be involved with treatment decisions as much as they want to be. Healthcare professionals should also be aware that it is important for patients to be heard and respected and that patients should always feel comfortable speaking up for themselves.

## Conclusions

The most important point that patients should take away from any discussion about biosimilars is that they are just as safe and effective as the reference product. Biosimilars have been through a rigorous regulatory process to ensure their efficacy and safety are highly similar to the reference product prior to their approval. In the case of interchangeable biosimilars, that also includes switching multiple times between the reference product and the biosimilar to confirm that switching does not impact their efficacy or safety. Biosimilars are intended to provide cost-effective treatment options and to provide more choice to patients. It is understandable that some patients may have concerns about whether biosimilars will work as well as the reference product and whether there will be any new side effects. To address these concerns, there is a need for more available information about biosimilars for both patients and healthcare professionals. All patients should be assured of the quality, safety, and effectiveness of taking biosimilars.

## Figures and Tables

**Table:** 

	Visuals	Text on screen	Voiceover script (2 min*)
1	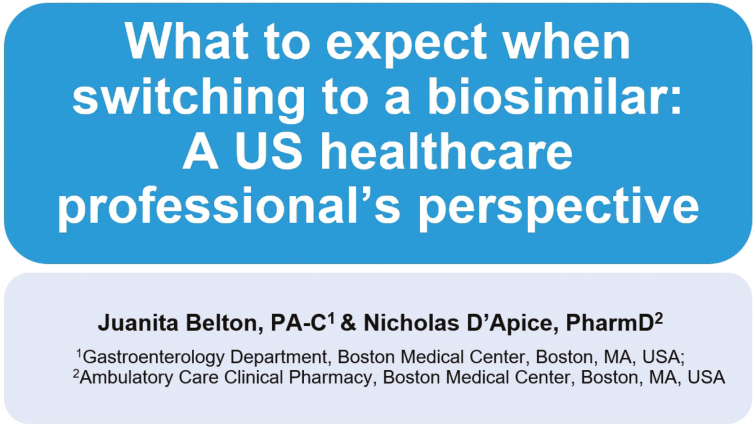	**What to expect when switching to a biosimilar: A US healthcare professional’s perspective** Juanita Belton, PA-C^1^ & Nicholas D’Apice, PharmD^2^ ^1^Gastroenterology Department, Boston Medical Center, Boston, MA, USA; ^2^Ambulatory Care Clinical Pharmacy, Boston Medical Center, Boston, MA, USA	What to expect when switching to a biosimilar: A healthcare professional’s perspective.
2	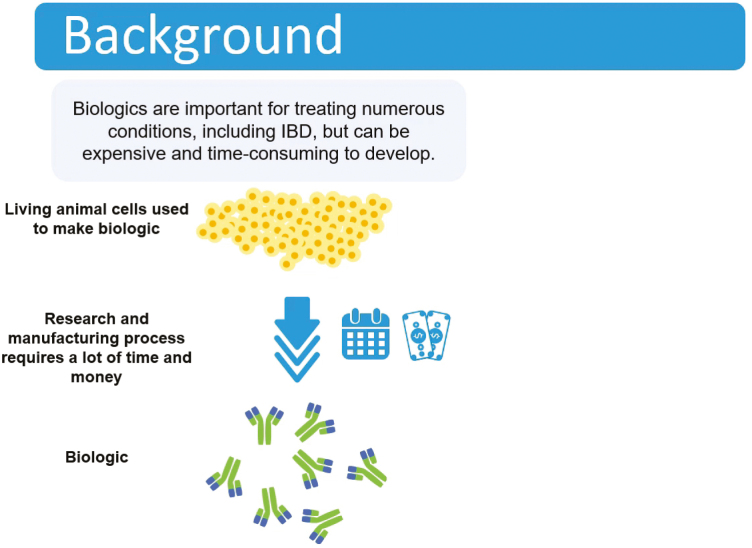	**Background** Biologics are important for treating numerous conditions, including IBD, but can be expensive and time-consuming to develop. Living animal cells used to make biologic Research and manufacturing process requires a lot of time and money Biologic	Biologic medications are large-molecule medications produced in living cells or tissues. Biologics have become increasingly important for treating a number of conditions, including autoimmune conditions such as IBD. However, their production requires a significant amount of time and cost.
3	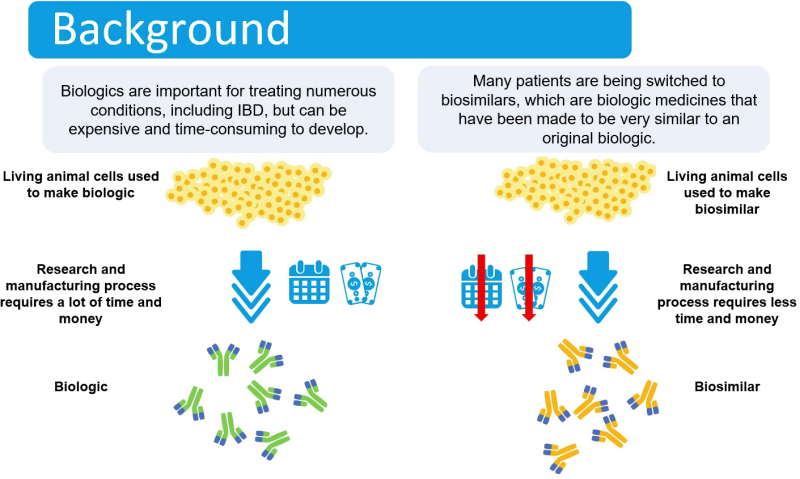	Many patients are being switched to biosimilars, which are biologic medicines that have been made to be very similar to an original biologic. Living animal cells used to make biosimilar Research and manufacturing process requires less time and money Biosimilar	Biosimilars are biologic medications that are made to be very similar to their original reference products. However, biosimilars require less time and cost to produce. Because of this, many patients may experience being switched to biosimilars.
4	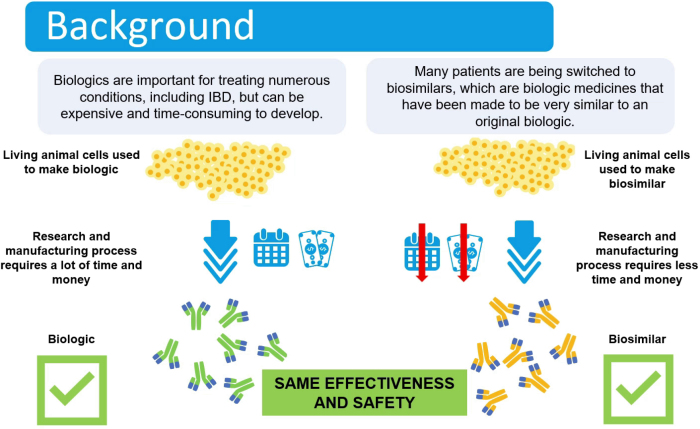	SAME EFFECTIVENESS AND SAFETY	Biosimilars are proven to have the same effectiveness and safety as their biologic reference product.
5	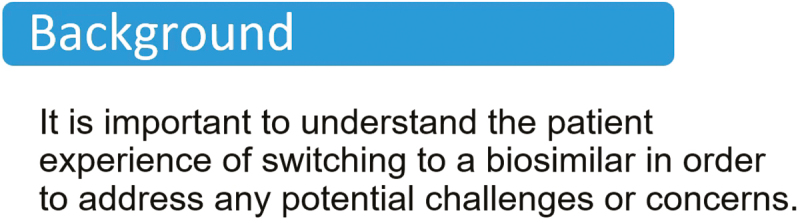	**Background** It is important to understand the patient experience of switching to a biosimilar in order to address any potential challenges or concerns.	As the switch to biosimilars becomes more common, it is important to understand the patient experience of being switched to a biosimilar medication, in order to address any potential challenges or concerns with switching.
6	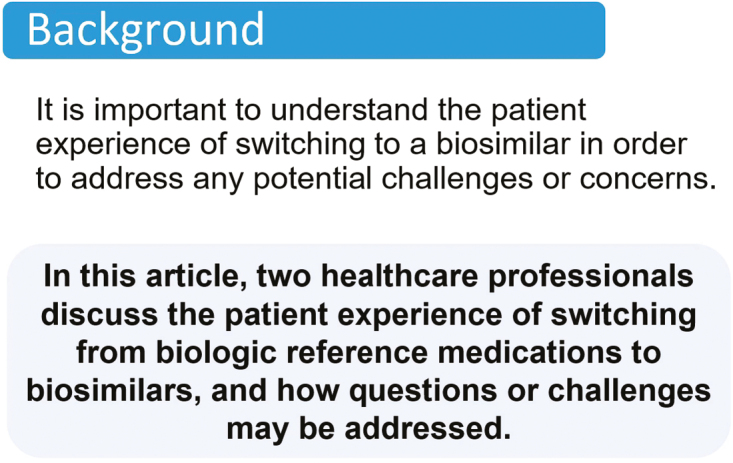	In this article, two healthcare professionals discuss the patient experience of switching from biologic reference medications to biosimilars, and how questions or challenges may be addressed.	In this article, a physician assistant and a pharmacist discuss the patient experience of switching to a biosimilar, and how to address any questions or challenges that may arise from the switch.
7	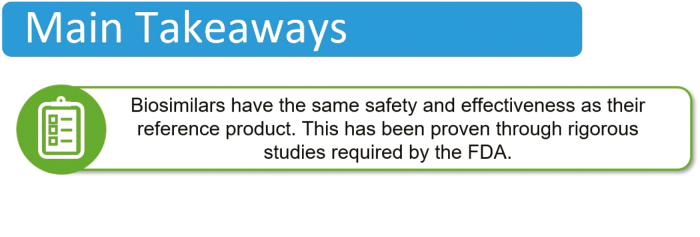	**Main Takeaways** Biosimilars have the same safety and effectiveness as their reference product. This has been proven through rigorous studies required by the FDA.	The main conclusions drawn from this discussion are as follows. First, healthcare professionals should emphasize to patients that biosimilars are proven to have the same effectiveness and safety as their reference products through rigorous testing required by the FDA.
8	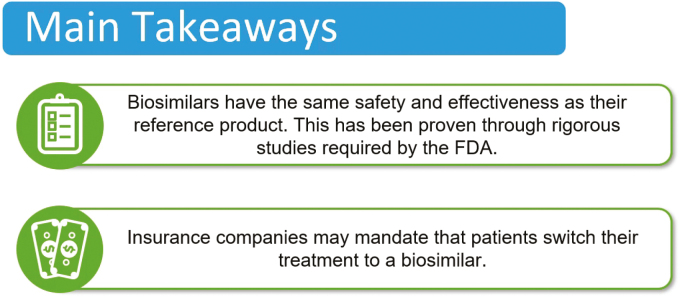	Insurance companies may mandate that patients switch their treatment to a biosimilar.	Second, it is important to explain to patients that they may be switched to biosimilars by their insurance company.
9	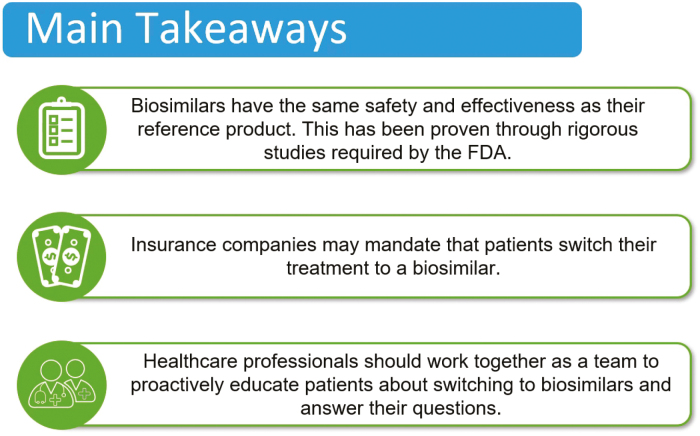	Healthcare professionals should work together as a team to proactively educate patients about switching to biosimilars and answer their questions.	Finally, it is important for healthcare professionals to work together as a team to proactively educate patients about the switch to a biosimilar and to answer any and all questions patients may have.
	(*End*)		

## Video Abstract

The findings of this article are also available as a playable video in the HTML version of this article.

(Spelling: English US)

*Per Words to Time; https://wordstotime.com/
